# Integrated analysis of microRNA and messenger RNA expression profiles reveals functional microRNA in infectious bovine rhinotracheitis virus-induced mitochondrial damage in Madin-Darby bovine kidney cells

**DOI:** 10.1186/s12864-024-10042-6

**Published:** 2024-02-08

**Authors:** Yingcai Ma, Xueping Guo, Qin He, Lu Liu, Zelong Li, Xiaomin Zhao, Wenxi Gu, Qi Zhong, Na Li, Gang Yao, Xuelian Ma

**Affiliations:** 1 College of Veterinary Medicine, Xinjiang Agricultural University, Urumqi, 830052 China; 2 Xinjiang key Laboratory of New Drug Study and Creation for Herbivorous Animal (XJ-KLNDSCHA), Xinjiang Agricultural University, Urumqi, 830052 China; 3 College of Veterinary Medicine, Northwest A & F University, Yangling, 712100 China; 4 Institute of Animal Science, Xinjiang Academy of Animal Sciences, Urumqi, 830011 China

**Keywords:** Infectious bovine rhinotracheitis virus (IBRV), MicroRNA (miRNA), miR-10a, miR-182, Mitochondrial damage

## Abstract

**Background:**

Studies have confirmed that Infectious bovine rhinotracheitis virus (IBRV) infection induces mitochondrial damage. MicroRNAs (miRNAs) are a class of noncoding RNA molecules, which are involved in various biological processes and pathological changes associated with mitochondrial damage. It is currently unclear whether miRNAs participate in IBRV-induced mitochondrial damage in Madin-Darby bovine kidney (MDBK) cells.

**Results:**

In the present study, we used high-throughput sequencing technology, Gene Ontology (GO) and Kyoto Encyclopedia of Genes and Genomes (KEGG) enrichment analysis to screen for mitochondria-related miRNAs and messenger RNAs (mRNAs). In total, 279 differentially expressed miRNAs and 832 differentially expressed mRNAs were identified in 6 hours (IBRV1) versus 24 hours (IBRV2) after IBRV infection in MDBK cells. GO and KEGG enrichment analysis revealed that 42 differentially expressed mRNAs and 348 target genes of differentially expressed miRNAs were correlated with mitochondrial damage, and the miRNA-mitochondria-related target genes regulatory network was constructed to elucidate their potential regulatory relationships. Among the 10 differentially expressed miRNAs, 8 showed expression patterns consistent with the high-throughput sequencing results. Functional validation results showed that overexpression of miR-10a and miR-182 aggravated mitochondrial damage, while inhibition of miR-10a and miR-182 alleviated mitochondrial damage.

**Conclusions:**

This study not only revealed the expression changes of miRNAs and mRNAs in IBRV-infected MDBK cells, but also revealed possible biological regulatory relationship between them. MiR-10a and miR-182 may have the potential to be developed as biomarkers for the diagnosis and treatment of IBRV. Together, Together, these data and analyses provide additional insights into the roles of miRNA and mRNA in IBRV-induced mitochondria damage

**Supplementary Information:**

The online version contains supplementary material available at 10.1186/s12864-024-10042-6.

## Background

Infectious bovine rhinotracheitis virus (IBRV) or bovine herpesvirus type-1 (BoHV-1), is a double-stranded DNA virus belonging to the family Herpesviridae, subfamily Alphaherpesvirinae [1]. IBRV infection causes various cattle diseases, such as rhinitis, encephalitis, conjunctivitis, and vulvovaginitis [2, 3]. It can also lead to abortion and immunosuppression, leading to more serious diseases. IBRV infection is a major cause of cattle mortality worldwide [4]. After IBRV infection, the virus initially replicates on mucosal surfaces, then spreads to the nervous system and stays in a latent infection state [5]. The ability of the virus in persistence and multiplication makes IBRV difficult to be eradicated in the infected animals [6, 7].

Mitochondrion is a membrane-bound organelle found in the cytoplasm of almost all eukaryotic cells, the primary function of which is to generate large quantities of energy in the form of adenosine triphosphate [8]. The structure and function of mitochondria are subject to multiple sources of damage [9, 10], in which viral infection plays a vital role. Viral infections destroy the dynamic balance of mitochondria [11, 12]. Following herpes simplex virus 1 (HSV-1) infection, the UL16 protein of HSV-1 couples with ANT2 in human umbilical vein endothelial cells and promotes the metabolism of cell mitochondria [13]. Classical swine fever virus (CSFV) infection can induce mitophagy via the CSFV nonstructural protein 5A, leading to loss of mitochondrial membrane potential (MMP) and mitochondrial fission and increasing the expression level of reactive oxygen species (ROS) [14]. In addition, studies have also shown that IBRV infection damaged mitochondrial function by affecting the expression of mitochondrial function-associated proteins and antioxidant enzymes in the mitochondrial respiratory chain (RC) complexes [15]. IBRV infection can induce loss of MMP, and reduces adenosine triphosphate production [16]. The decrease of MMP may lead to the opening of mitochondrial permeability transition pore (mPTP) and increase the generation of ROS [17]. Our previous research has found that IBRV infection of MDBK cells induces depolarization of the MMP and opening of mPTP [18].

MicroRNAs (miRNAs) are a class of single-stranded noncoding small-molecule RNAs with a length of 21–25 nucleotides that exist in most eukaryotes [19, 20]. They are involved in a variety of biological processes, such as inflammation, cell proliferation and apoptosis, and play an important role in antiviral response [21, 22]. More and more evidence shows that viruses modulate cellular miRNA expression profiles during viral infections to facilitate or inhibit viral replication [23]. In addition, miRNAs have been intricately linked with mitochondria, both directly and indirectly [24, 25]. A previous study showed that miR-302b and miR-372 were upregulated in human cell lines after Sendai virus infection, and miR-302b and miR-372 were involved in dynamin-related protein 1 (DRP1)-dependent mitochondrial fragmentation and disruption of mitochondrial metabolism by attenuating solute carrier family 25 member 12 [26]. MiR-aU14 encoded by Human herpesvirus 6A selectively inhibited the processing of multiple miR-30 family members by direct interaction with the respective pri-miRNA hairpin loops and subsequent loss of miR-30 and activation of the miR-30-p53-DRP1 axis triggered profound disruption of the mitochondrial architecture [27]. Our previous research has found that IBRV infection of MDBK cells induces apoptosis and mitochondrial dysfunction [18], But whether miRNAs participate in IBRV-induced mitochondrial damage of MDBK cells remains unclear. Therefore, this study used high-throughput sequencing technology to screen miRNAs differentially expressed in MDBK cells infected with IBRV and mock group, and the impact of differentially expressed miRNAs on mitochondria function was verified to provide a theoretical basis for further elucidating the pathogenesis of IBR and new molecular targets for research and development of new drugs targeting IBRV.

## Results

### High-throughput sequencing data summary

Our previous study proved that IBRV infection could induce mitochondrial damage in MDBK cells, abnormal opening of the mPTP of MDBK cells, and MMP depolarization. To determine miRNA expression changes in MDBK cells before and after infection with IBRV, we used high-throughput sequencing to analyze IBRV-induced miRNA expression profile changes. In total, 13.09, 11.06, 11.40, 11.08, 9.66.7, 11.66, 11.46, 8.76, and 9.13 million high-quality clean tags of miRNAs were obtained from the Mock groups (Mock-1, Mock-2, and Mock-3), 6 hours (IBRV1-1, IBRV1-2, and IBRV1-3) and 24 hours (IBRV2-1, IBRV2-2, IBRV2-3), respectively. The miRNA sequence quality control results are shown in Additional file 1: Table S1.

### Heatmap and cluster analysis of differentially expressed miRNAs and mRNAs

**Fig. 1:**
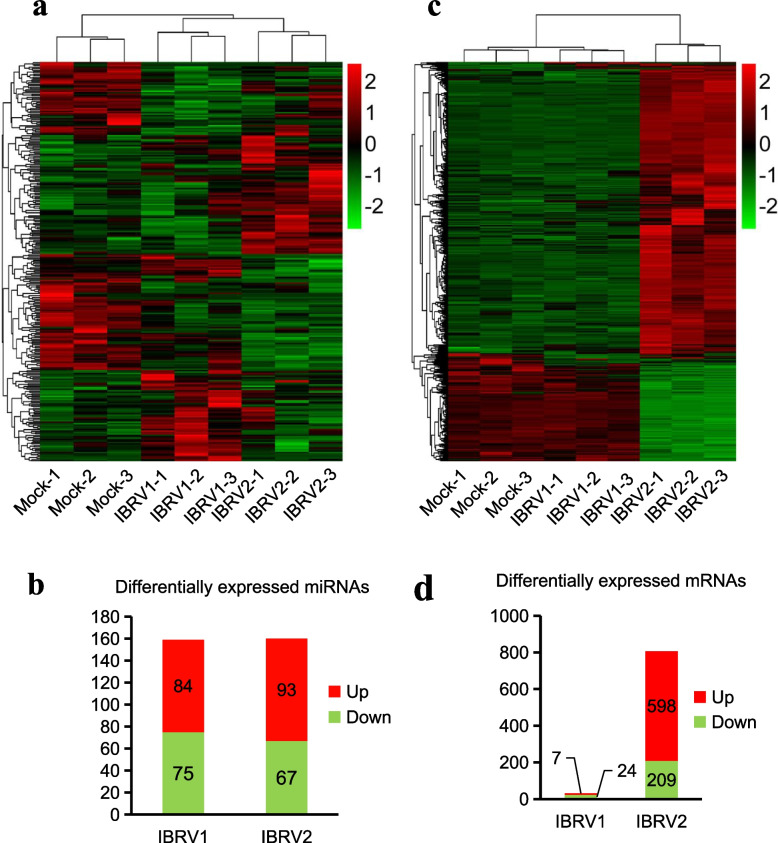
Clustering and heatmap analysis of differentially expressed microRNAs (miRNAs) and messenger RNAs (mRNAs). **a** Clustering and heatmap analysis of differentially expressed miRNAs. **b** including 84 upregulated mRNAs and 75 downregulated mRNAs in the IBRV1 group and 93 upregulated mRNAs and 67 downregulated mRNAs in the IBRV2 group. **c** Clustering and heatmap analysis of differentially expressed mRNAs. **d** including 7 upregulated mRNAs and 24 downregulated mRNAs in the IBRV1 group and 598 upregulated mRNAs and 209 downregulated mRNAs in the IBRV2 group

We obtained 159 differentially expressed miRNAs (*p* < 0.05) in IBRV1 group, of which 84 differentially expressed miRNAs were upregulated and 75 differentially expressed miRNAs were downregulated. There were 160 differentially expressed miRNAs (*p* < 0.05) in IBRV2 group, of which 93 differentially expressed miRNAs were upregulated, and 67 differentially expressed miRNAs were downregulated (Fig. 1a, b). We obtained 31 differentially expressed mRNAs (*p* < 0.05), with a fold change of > 2 (log2 fold-change > 1 or < -1) in IBRV1 group, of which 7 differentially expressed mRNAs were upregulated and 24 differentially expressed mRNAs were downregulated. In IBRV2 group we obtained 807 differentially expressed mRNAs (*p* < 0.05) with a fold change of > 2 (log2 fold-change > 1 or < -1) in which 598 differentially expressed mRNAs were upregulated, and 209 differentially expressed mRNAs were downregulated (Fig. 1c, d). In total 279 differentially expressed miRNAs (Additional file 2: Table S2) and 832 differentially expressed mRNAs (Additional file 3: Table S3) were identified in the IBRV1 group versus IBRV2 group.

### GO and KEGG enrichment analysis of differentially expressed mRNAs

**Fig. 2:**
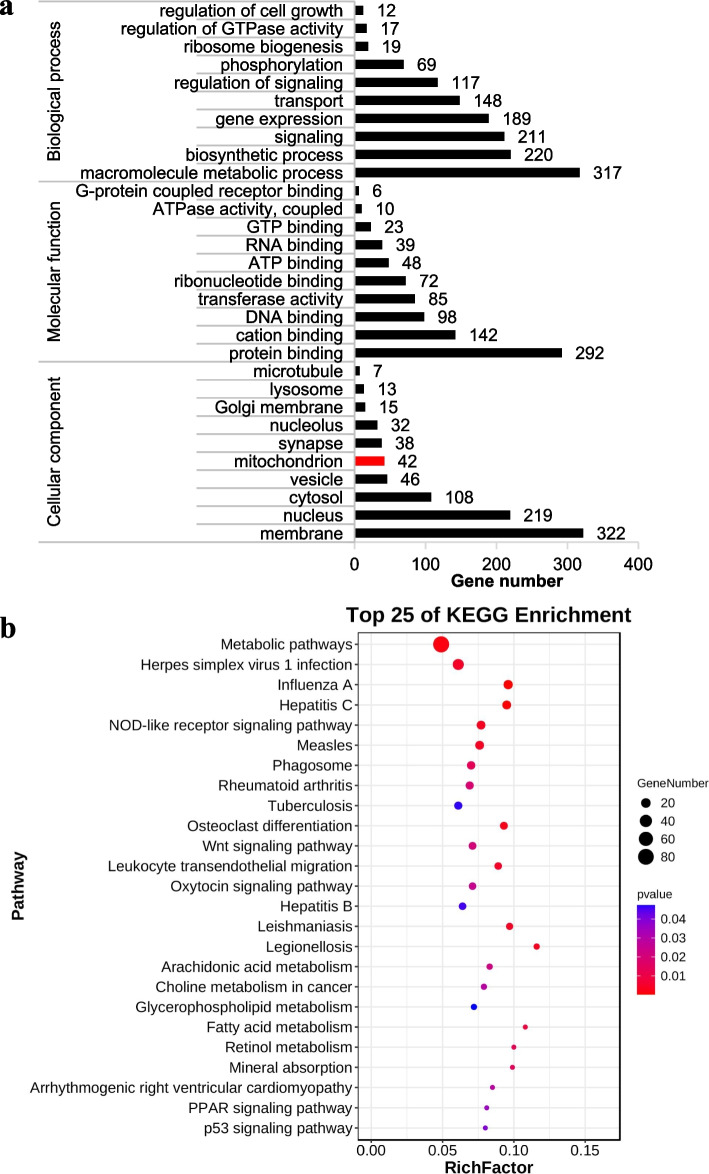
Gene Ontology (GO) and Kyoto Encyclopedia of Genes and Genomes (KEGG) enrichment analysis of differentially expressed messenger RNAs (mRNAs). **a** GO enrichment analysis of 832 differentially expressed mRNAs. **b** Top 25 what in the KEGG pathway analysis of 832 differentially expressed mRNA. The degree of KEGG enrichment was assessed by the richness factor, *p*-value, and gene number. The closer the* p*-value was to zero, the greater the richness factor was. The greater the gene number was, the more significant the enrichment was

A total of 832 differentially expressed mRNAs were searched in the GO (http://www.geneontology.org) and KEGG (http://www.genome.jp/kegg/) database enrichment analysis. According to the GO enrichment analysis, these differentially expressed mRNAs were mostly involved in the BP (e.g., macromolecule metabolic process, biosynthetic process, signaling, and gene expression), MF (e.g., protein binding, DNA binding, transferase activity, and ATP binding), and CC (membrane, cytosol, and mitochondrion) domains in which 42 differentially expressed mRNAs were enriched in the CC mitochondrion (Fig. 2a, Additional file 4: Table S4). The KEGG pathway analysis showed that these differentially expressed mRNAs were mostly involved in HSV-1 infection and the nucleotide-binding oligomerization domain (NOD)-like receptor, Wnt, oxytocin, peroxisome proliferator-activated receptor (PPAR), and p53 signaling pathways (Fig. 2b, Additional file 5: Table S5).

### Target genes prediction of differentially expressed miRNAs and integrative analysis

**Fig. 3:**
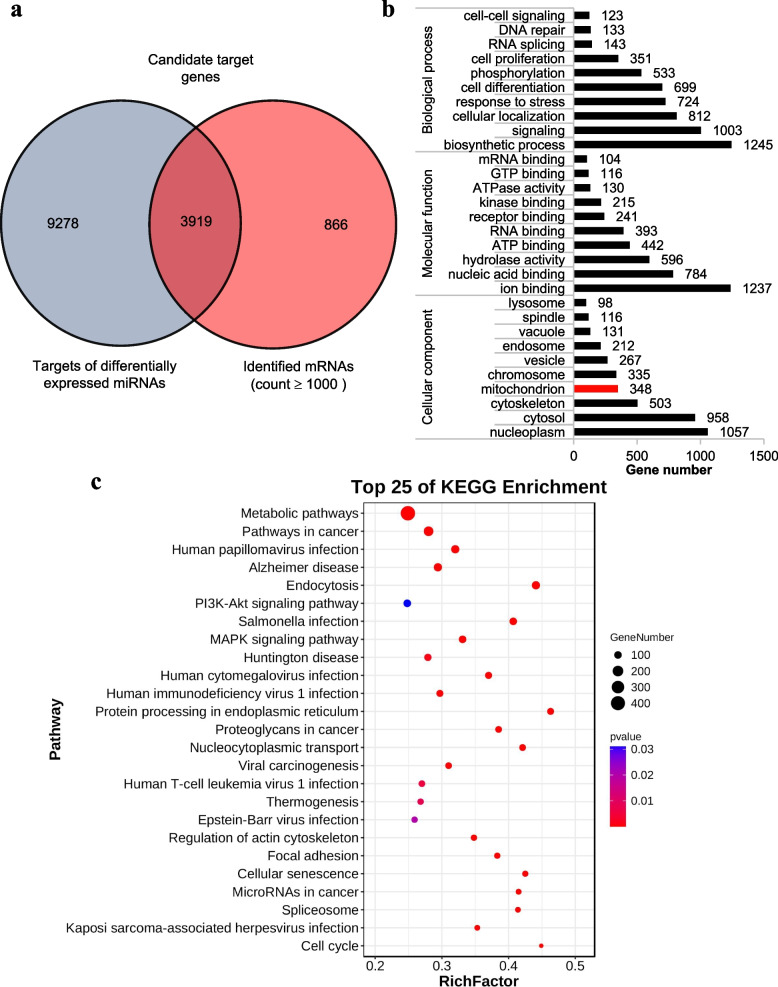
Gene Ontology (GO) and Kyoto Encyclopedia of Genes and Genomes (KEGG) enrichment analysis of target genes of differentially expressed microRNAs (miRNAs). **a** The intersection (candidate target genes) of the target genes of the differentially expressed miRNAs and identified mRNAs (count ≥ 1000). **b** GO enrichment analysis of 3,919 candidate target genes. **c** Top 25 what in the KEGG pathway analysis of the 3,919 candidate target genes. The degree of KEGG enrichment was assessed by the richness factor, *p*-value, and gene number. The closer the* p*-value was to zero, the greater the richness factor was. The greater the gene number was, the more significant the enrichment was

To predict the interaction between miRNAs and mRNAs during IBRV infection, in total 13,197 predicted target genes of 279 differentially expressed miRNAs were obtained via RNAhybrid (v2.1.2) + svm_light (v6.01), Miranda (v3.3a), and TargetScan (v7.0), as shown in Additional file 6: Table S6. Combined with the mRNAs, the predicted target genes and 4,785 identified mRNAs (count ≥ 1,000) were intersected (Fig. 3a). A total of 3,919 candidate target genes with 279 differentially expressed miRNAs were obtained in the IBRV1 group versus IBRV2 group (Additional file 7: Table S7).

### Candidate target gene GO and KEGG enrichment analysis

The GO enrichment analysis revealed that 3,919 candidate target genes were mostly involved in BP (e.g., biosynthetic process, signaling, and cellular localization), MF (e.g., ion binding, nucleic acid binding, and ATP binding), and CC (e.g., nucleoplasm, cytosol, and mitochondrion). Of these candidate target genes, 348 genes were enriched in the CC mitochondrion (Fig. 3b, Additional file 8: Table S8). The KEGG pathway analysis showed that these target genes were mostly involved in the phosphatidylinositol-3-kinase (PI3K)/protein kinase B (Akt) signaling pathway, mitogen-activated protein kinases (MAPK) signaling pathway, protein processing in the endoplasmic reticulum, nucleocytoplasmic transport, viral carcinogenesis, Epstein-Barr virus infection, cellular senescence, Kaposi sarcoma-associated HSV infection, and cell cycle pathways (Fig. 3c, Additional file 9: Table S9).

### miRNA-mitochondria-related target genes regulatory networks

The miRNA-mitochondria-related target genes regulatory network contained 273 differentially expressed miRNAs (122 upregulated and 151 downregulated) and 348 mitochondria-related target genes (Additional file 10: Figure S1; Additional file 11: Table S10). For some differentially expressed miRNAs, such as miR-1307-z and miR-574-y, only one target gene was identified. However, most of the differentially expressed miRNAs targeted on several genes. In addition, several miRNAs targeted on one gene. For example, bta-let-7a-5p targeted on 19 different mRNAs, bta-miR-10162-5p targeted on 48 different mRNAs, and bta-miR-10a targeted on 27 different mRNAs. TGM2 was targeted by 64 miRNAs, ACACB was targeted by 61 miRNAs, and ACO2 was targeted by 40 miRNAs.

### Validation of miRNAs by reverse transcription-quantitative polymerase chain reaction (RT-qPCR)

**Fig. 4:**
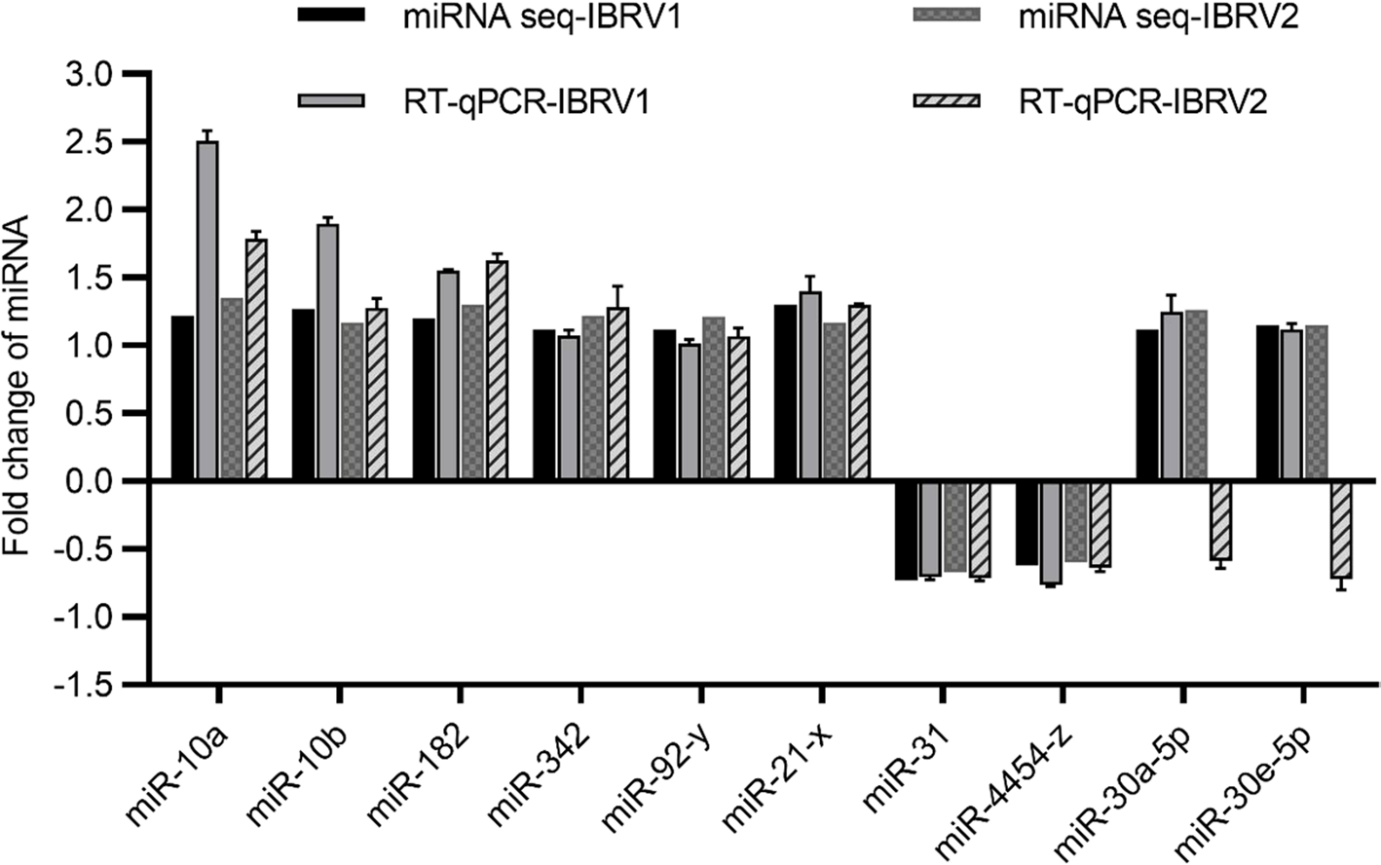
RT-qPCR validation of selected miRNAs. RT-qPCR results for 10 miRNAs (bta-miR-10a, bta-miR-10b, bta-miR-182, bta-miR-342, miR-92-y, miR-21-x, bta-miR-31, miR-4454-z, bta-miR-30a-5p, and bta-miR-30e-5p). All data were calculated using the 2^−ΔΔCT^ method, and the miRNA level of each sample was normalized according to U6 expression

To validate the miRNA expression profile obtained from sequencing, we randomly selected 10 differentially expressed miRNAs for stem-loop RT-qPCR. The expression patterns of 8 of 10 miRNAs were consistent with those of the miRNA high-throughput sequencing results (Fig. 4). Although the relative expression level of bta-miR-30a-5p and bta-miR-30e-5p were not always completely consistent, the results of the RT-qPCR analysis confirmed the reliability and accuracy of the miRNA sequencing data.

### miRNA (miR-10a and miR-182)-mitochondria-related target genes regulatory networks

**Fig. 5:**
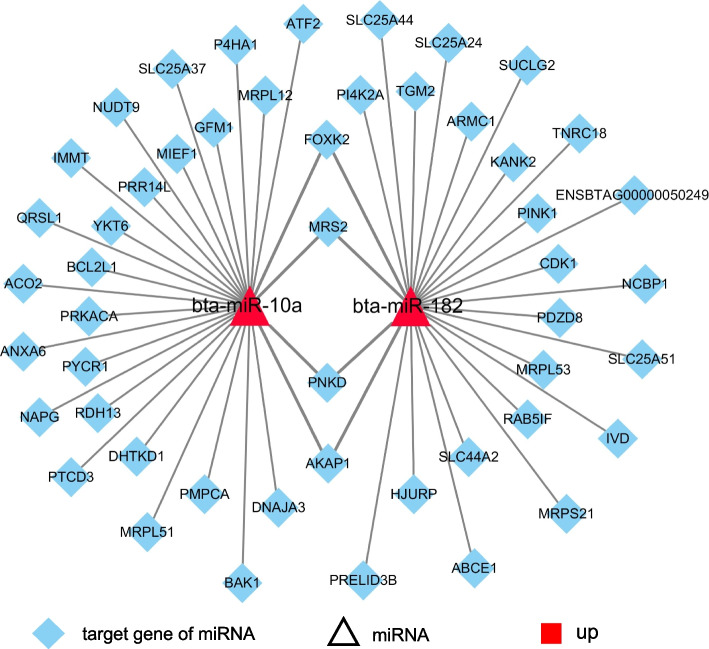
MiRNA(miR-10a and miR-182)-mitochondria-related target gene regulatory networks. The triangles represent miRNA, the diamonds represent the target gene of the miRNAs, and the red and green shaded represent upregulated and downregulated miRNAs, respectively

Based on the detection of miRNA expression in MDBK cells, miR-10a and miR-182 were highly expressed and were selected for subsequent study. MiRNA (miR-10a and miR-182)-mitochondria-related target genes regulatory network contained 2 differentially expressed and upregulated miRNAs, and 54 mitochondria-related target genes (Fig. 5, Additional file 12: Table S11). Among, miR-10a targeted on 28 different mRNAs, miR-182 targeted on 26 different mRNAs. FOXK2, MRS2, PNKD and AKAP1 were targeted by miR-10a and miR-182.

### Functional validation of miR-10a and miR-182

**Fig. 6:**
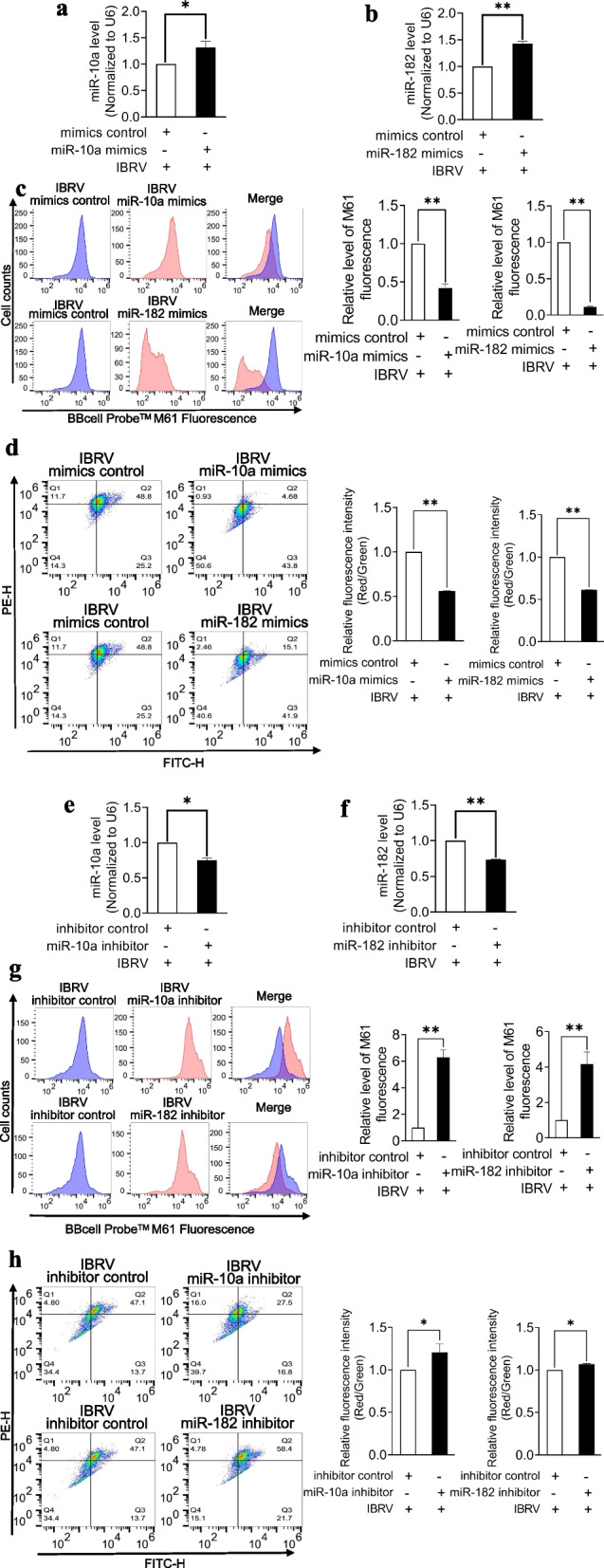
Functional validation of miR-10a and miR-182. **a** Expression levels of miR-10a after MDBK cells were transfected with miR-10a mimics. **b** Expression levels of miR-182 after MDBK cells were transfected with miR-182 mimics. **c** Changes of mitochondrial permeability transition pore (mPTP) opening after MDBK cells were transfected with miR-10a and miR-182 mimics. **d** Changes in the mitochondrial membrane potential (MMP) after MDBK cells were transfected with miR-10a and miR-182 mimics. **e** Expression levels of miR-10a after MDBK cells were transfected with miR-10a inhibitor. **f** Expression levels of miR-182 after MDBK cells were transfected with miR-182 inhibitor. **g** Changes of mitochondrial permeability transition pore (mPTP) opening after MDBK cells were transfected with miR-10a and miR-182 inhibitor. **h** Changes in the mitochondrial membrane potential (MMP) after MDBK cells were transfected with miR-10a and miR-182 inhibitor. *: *p* < 0.05, **: *p* < 0.01

To validate the function of miR-10a and miR-182, we transfected MDBK cells with miRNA mimics or miRNA inhibitors for functional studies. The results revealed that miR-10a (*p* < 0.05) and miR-182 expression (*p* < 0.01) were significantly increased in MDBK cells post-transfection with the miR-10a and miR-182 mimics (Fig. 6a, b) compared with the control group, and the miR-10a and miR-182 mimics promoted mPTP opening and MMP depolarization (*p* < 0.01). (Fig. 6c, d). Conversely, miR-10a (*p* < 0.05) and miR-182 expression (*p* < 0.01) were significantly decreased in MDBK cells post-transfection with the miR-10a and miR-182 inhibitor (Fig. 6e, f) compared with the control group, and mPTP opening (*p* < 0.01) and MMP depolarization (*p* < 0.05) were rescued by the miR-10a and miR-182 inhibitor (Fig. 6g, h).

## Discussion

IBR mainly leads to cattle’s fever, abortion, stillbirth, and neurological disease. It is one of the main pathogens resulting in respiratory disease syndrome that causes huge economic losses in the cattle industry [28]. External stimulus can readily damage mitochondrial morphology and function. Viral infections are an important factor of mitochondrial damage [12]. The miRNA affects various physiological mechanisms of cells by regulating their target genes [29]. The miRNA expression profile is also profoundly affected by viral infection [30]. For example, 619 unique miRNAs were identified in cats infected with feline parvovirus and dogs infected with canine parvovirus [31]. Thirteen differentially expressed miRNAs were identified in variant pseudorabies virus-infected PK15 cells, among which 8 miRNAs were upregulated and 5 miRNAs were downregulated [32]. In the present study, 279 differentially expressed miRNAs and 832 differentially expressed mRNAs were identified in IBRV-infected MDBK cells, which have a similar change trend to the previous studies. Furthermore, research revealed that miRNA is involved in the process of mitochondrial damage induced by virus [33, 34].

In this study, KEGG enrichment analysis indicated that 832 differentially expressed mRNAs were mainly involved in metabolic, NOD-like receptor, Wnt, PPAR, and p53 signaling pathways and in glycerophospholipid metabolism. The target genes of the differentially expressed miRNAs were mainly involved in endocytosis, the PI3K-Akt signaling pathway, nucleocytoplasmic transport, MAPK signaling pathway, and viral carcinogenesis pathways, among these, the NOD-like receptor, Wnt, PPAR, PI3K-Akt, and MAPK signaling pathways were closely related to mitochondrial function [35–37]. For example, NOD-like receptor family member X1 (NLRX1) localized to the mitochondrial outer membrane. As reported previously, overexpression of NLRX1 can induce the generation of ROS and mediate the antiviral activity of Mitochondrial antiviral signaling (MAVS) by directly interacting with MAVS [38, 39]. The Wnt signaling pathway plays an important role in the regulation of the cellular nonautonomous mitochondrial stress response [40]. The PPAR signaling pathway is associated with cellular injury and mitochondrial energy metabolism disorders in diabetic cardiomyopathy [41]. Our study suggests that these mitochondria-related differentially expressed mRNAs and target genes of differentially expressed miRNAs may play important roles in mitochondrial damage induced by IBRV.

Current research reported that miR-10a and miR-182 play the essential roles in various cancers and viral infections [42, 43]. The miR-10a inhibits proliferation and migration and promotes apoptosis of breast cancer cells via phosphoinositide/Akt/mammalian target of rapamycin (mTOR) signaling, and the mitochondrial apoptotic pathway [44], and miR-10a is downregulated by DNA methylation and functions as a tumor suppressor in gastric cancer cells [45]. In addition, the miR-10a duplex significantly upregulates the biosynthesis of Coxsackievirus B3 (CVB3) and positively modulates gene expression. Furthermore, the rare allele T in the pri-miR-10a coding region may be involved in the pathogenesis of acute viral myocarditis caused by CVB3 by weakening the host antivirus immune response [46, 47]. A member of the miR-183 cluster (miR-183, miR-182, miR-96), miR-182 can promote cancer invasion by linking rearranged during transfection (RET) oncogene-activated nuclear factor κ-light-chain-enhancer of activated B cells (NF-κB) to loss of the hes family bHLH transcription factor 1 (HES1)/notch receptor 1 (NOTCH1) regulatory circuit [48]. In addition, miR-182 can inhibit human cytomegalovirus replication through activation of the type I interferon (IFN-I) response by targeting Forkhead box O3 (FOXO3) in neural cells [49]. Gao et al. reported that miR-182 may be involved in the regulation of mitochondrial function [50]. It has also been shown that miR-182 can protect mitochondrial structure by acting with corticosteroids to reduce mitochondrial disruption, and mitigate neuroinflammation and cell death after ischemic stroke [51]. However, no studies have investigated whether miR-10a and miR-182 participate in IBRV-induced mitochondrial damage.

In the present study, we obtained a total of 1,140 and 1,368 target genes of miR-10a and miR-182 by miRNA target gene prediction, respectively. Among these, 52 and 59 target genes of miR-10a and miR-182 were localized in mitochondria. By overexpressing and inhibiting miR-10a and miR-182, we found that both miR-10a and miR-182 are involved in IBRV-induced mitochondrial damage in MDBK cells. The regulatory mechanisms of mir-10a and mir-182 on IBRV induced mitochondrial damage need to be further studied.

## Conclusions

This study not only revealed the expression changes of miRNAs and mRNAs in IBRV-infected MDBK cells, but also revealed possible biological regulatory relationship between them. MiR-10a and miR-182 may have the potential to be developed as biomarkers for the diagnosis and treatment of IBRV. Together, Together, these data and analyses provide additional insights into the roles of miRNA and mRNA in IBRV-induced mitochondria damage.

## Materials and methods

### Cells, virus, and primers

In the present study, MDBK (NBL-1) cells, purchased from the cell bank of the Chinese Academy of Sciences (Shanghai, China). were cultured in Dulbecco’s Modified Eagle Medium (DMEM) (Biological Industries, Beit Haemek, Israel) supplemented with 100 IU of penicillin and 100 mg of streptomycin per milliliter at 37℃ in an incubator with 5% CO_2_. All experiments were performed with cells between passage 4 and 8. IBRV AV21 strain was purchased from the China Institute of Veterinary Drug Control (Beijing, China). Primers, mimics, and inhibitor of miRNAs were synthesized by Shanghai Sangon Biological Engineering Technology Company Limited (Shanghai, China). The sequences are shown in Additional file 13: Table S12.

### Sample preparation

Our previous study have reported that the mitochondrial damage began at 6 h of IBRV infection and was most obvious at 24 h [18]. Therefore, we selected two time points of 6 h and 24 h for following study. MDBK cells were seeded at a density of 3 × 10^6^ cells per plate into nine 100 mm culture dishes.MDBK cells were divided into three experimental groups, i.e., Mock group with 3 duplications (Mock-1, Mock-2, and Mock-3), IBRV infection for 6 hour group (IBRV1) with 3 duplications (IBRV1-1, IBRV1-2, and IBRV1-3) and IBRV infection for 24 hour group (IBRV2) with 3 dupications (IBRV2-1, IBRV2-2, and IBRV2-3). The IBRV-infected groups were inoculated with 1.5 multiplicity of infection (MOI) virus when the cells reached confluence (85%). The same volume of serum-free DMEM was added to the Mock groups, and the cells from these groups were collected 6 hours and 24 hours later. Then, 1 mL of TRIzol (Invitrogen, Carlsbad, CA, USA) was added, and the cells were stored at -80°C after liquid nitrogen flash freezing.

### High-throughput sequencing

High-throughput sequencing was performed by Gene Denovo Biotechnology Co. (Guangzhou, China). After total RNA was extracted using a TRIzol reagent kit (Invitrogen, Carlsbad, CA, USA), RNA molecules in a size range of 18–30 nt were enriched by polyacrylamide gel electrophoresis. Then, 3′ adapters were added, and 36–44 nt RNAs were enriched. Subsequently, 5′ adapters were ligated to the RNAs. The ligation products were reverse transcribed by polymerase chain reaction (PCR) amplification, and 140–160 bp size PCR products were enriched to generate a complementary DNA (cDNA) library. After total RNA was extracted, eukaryotic mRNA was enriched by Oligo (dT) beads (Invitrogen, Carlsbad, CA, USA). The enriched mRNA was then fragmented into short fragments using fragmentation buffer and reverse transcribed into cDNA using the NEBNext Ultra RNA Library Prep Kit for Illumina (NEB, Ipswich, MA, USA). The purified double-stranded cDNA fragments were end repaired, a base added, and ligated to Illumina sequencing adapters. The ligation reaction was purified with AMPure XP Beads (1.0X) (Beckman Coulter, USA) and PCR amplified. The resulting cDNA library was sequenced using Illumina Novaseq 6000 (Guangzhou, China).

### Alignment of miRNAs and mRNAs

The low-quality reads were removed to obtain clean tags, which were aligned with small RNAs in the GeneBank database and Rfam database to identify and remove ribosomal RNAs, small cytoplasmic RNAs, small nucleolar RNAs, small nuclear ribonucleic acids, and transfer RNA. The rest of the clean tags were then aligned with the ARS-UCD1.2 reference genome and searched against the miRBase database to identify existing miRNAs. Unmapped miRNAs were aligned with other species. All the unannotated tags were aligned with the ARS-UCD1.2 reference genome. According to the genome positions and hairpin structures predicted by miRDeep2 software (https://github.com/rajewsky-lab/mirdeep2), novel miRNA candidates were obtained. An index of the reference genome was built, and paired-end clean reads were mapped to the ARS-UCD1.2 reference genome using HISAT2 (v2.0.4).

### Quantification of miRNA and mRNA abundance

The miRNA expression level was calculated and normalized to transcripts per million (TPM) using the following formula:

$$TPM=\frac{T{10}^{6}}{N}$$

where T is the actual miRNA count, and N is the total count of clean tags (existing, known, and novel miRNAs).

The mRNA expression level was calculated and normalized to fragment per kilobase of transcript per million mapped reads (FPKM) using the formula below:

$$FPKM=\frac{{10}^{6}C}{NL/{10}^{3}}$$

where RPKM is the expression level of a given gene A, C is the number of fragments that are uniquely aligned to gene A, N is the total fragment number that is aligned to the reference gene, and L is the number of bases of the coding region of gene A.

### Prediction of miRNAs targets

RNAhybrid (v2.1.2) + svm_light (v6.01), Miranda (v3.3a), and TargetScan (v7.0) were used to predict targets of the miRNAs. The intersection of the target genes of the differentially expressed miRNAs and identified mRNAs was chosen as candidate targets of miRNAs.

### GO and KEGG enrichment analysis of differentially expressed miRNAs and mRNAs

Functional annotation was performed using GO (http://www.geneontology.org/) enrichment analysis and KEGG (http://www.genome.ad.jp/kegg/) pathway analysis. We performed functional annotation of differentially expressed mRNAs and target genes of differentially expressed miRNAs to cellular component (CC), molecular function (MF), and biological process (BP) using the GO database. In the enrichment analysis, the gene number of gene sets in each GO term was counted. For the KEGG analysis, the top 25 entries in the KEGG pathways were exhibited, The corrected p-value threshold was *p* ≤ 0.05.

### miRNA-mitochondria-related target gene regulatory networks

The miRNA-mitochondria-related target gene regulatory networks were constructed using a combination of miRNA-mitochondria-related target gene pairs, and the regulatory networks were visualized using Cytoscape (v3.7.0) (http://www.cytoscape.org/).

### RT-qPCR

Ten miRNAs were chosen from differentially expressed miRNAs using a simple random sampling method generated using Microsoft Office Excel (Microsoft Office Excel 2016, Microsoft Corporation, Redmond, USA). TRIzol (Invitrogen, Carlsbad, CA, USA) was used to extract total RNA from the Mock and IBRV groups. RNA was reverse-transcribed using the Hifair^®^ III 1st Strand cDNA Synthesis Kit (gDNA digester plus) (Yeasen, Shanghai, China), in accordance with the manufacturer’s protocol. cDNA was amplified by the 7500 Fast Real-Time PCR System (Applied Biosystems, Foster City, CA, USA) using 2x RealStar Green Fast Mixture with ROX II (GenStar, Beijing, China). All data were calculated using the 2^−ΔΔCt^ method, and the miRNA level of each sample was normalized according to U6 expression. Each group comprised three duplicate wells.

### Transfection of miRNAs

To confirm whether miR-10a and miR-182 played a role in mitochondrial damage, we investigated the biological functions of miR-10a and miR-182 in MDBK cells by overexpression and inhibition. The miRNA mimics and miRNA inhibitor were transfected into MDBK cells to overexpress or inhibit the expression of miR-10a and miR-182. The transfection efficiency of miR-10a and miR-182 was verified by RT-qPCR, and the biological functions of miR-10a and miR-182 were verified by flow cytometry. Mimics, inhibitors, and negative control oligonucleotides of miR-10a and miR-182 were purchased from Sangon Biotech Co., Ltd (Shanghai, China). MDBK cells were cultured to 60–70% confluence after being seeded onto six-well plates. Transfection of cells with oligonucleotides was performed using Lipofectamine 3000 reagent (Invitrogen, Carlsbad, CA, USA) at a final concentration of 100 nM in line with the manufacturer’s instructions. Twelve hours later, the transfected cells were inoculated with 1.5 MOI IBRV. After 6 and 24 hours of inoculation, the cells were harvested for further study.

### Determination of intracellular mPTP levels

The generation of mPTP was determined according to the manufacturer’s instructions using an mPTP assay kit (BestBio, Shanghai, China). Each sample was treated with 5 μl of BbcellProbe^TM^ M61 probe (200 μM) for 15 minutes and quencher for 15 minutes at 37°C. Opening of mPTP results in a decrease of fluorescence. Fluorescence was measured by flow cytometry (Mindray, Shenzhen, China), with 10,000 events collecting.

### Determination of intracellular MMP levels

The MMP was determined using a JC-1 mitochondrial membrane potential assay kit (Beyotime, Jiangsu, China). MDBK cells were washed with serum-free DMEM and Each sample incubated in JC-I working solution for 20 minutes in the dark at 37°C. After washing, the cells were re-suspended with JC-1 dye buffer, and JC-1 monomer fluorescence distribution and j-aggregates were measured, the fluorescence was measured by flow cytometry(Mindray, Shenzhen, China), with 10,000 events collecting. Mitochondrial depolarization of MDBK cells in the Mock and IBRV groups were calculated by a decrease in the red/green fluorescence intensity ratio. JC-1 monomers (green fluorescence) and JC-1 aggregates (red fluorescence) represent a low MMP and high MMP, respectively. Therefore, mitochondrial depolarization is indicated by a decrease in the red/green fluorescence intensity ratio.

### Statistical analysis

The data are presented as the means ± standard error of mean (SEM). Statistical comparisons were performed using an unpaired Student’s t-test. the statistical significance was evaluated by Graphpad Prism 8.0 software. Relative to the control, * *p* < 0.05 indicates significant difference and ** *p* < 0.01 indicates a highly significant difference.

### Supplementary Information

**Additional file 1:** **Table S1.** The miRNA sequence quality control results.

**Additional file 2:** **Table S2.** Differentially expressed miRNAs.

**Additional file 3:** **Table S3.** Differentially expressed mRNAs.

**Additional file 4:** **Table S4.** GO enrichment analysis of differentially expressed mRNAs.

**Additional file 5:** **Table S5.** KEGG enrichment analysis of differentially expressed mRNAs.

**Additional file 6:** **Table S6**. Target genes of differentially expressed miRNAs.

**Additional file 7:** **Table S7.** Candidate target genes of differentially expressed miRNAs.

**Additional file 8:** **Table S8.** GO enrichment analysis of target genes of differentially expressed miRNAs.

**Additional file 9:** **Table S9.** KEGG enrichment analysis of target genes of differentially expressed miRNAs.

**Additional file 10:** **Figure S1.** MiRNA-mitochondria-related target gene regulatory networks.

**Additional file 11:** **Table S10.** MiRNA-mitochondria-related target gene regulatory networks.

**Additional file 12:** **Table S11.** MiRNA (bta-miR-10a and bta-miR-182)-mitochondria-related target gene regulatory networks.

**Additional file 13:** **Table S12.** Primers, mimics and inhibitor of miRNAs.

**Additional file 14:** **Figure S1.** MiRNA-mitochondria-related target gene regulatory networks. The triangles represent miRNA, the diamonds represent the target gene of the miRNAs, and the red and green shaded represent upregulated and downregulated miRNAs, respectively.

## Data Availability

The datasets presented in this study can be found in online repositories. The names of the repository/repositories and accession numbers can be found below: https://www.ncbi.nlm.nih.gov/bioproject/PRJNA941037. Data generated during analysis are included in the manuscript as supplementary fles.

## Abbreviations

miRNA: MicroRNA
mRNA: Messenger RNA
IBRV: Infectious bovine rhinotracheitis virus
MMP: Mitochondrial membrane potential
ROS: Reactive oxygen species
mPTP: Mitochondrial permeability transition pore
GO: Gene ontology
KEGG: Kyoto Encyclopedia of Genes and Genomes
DRP1: Dynamin-related protein 1
NLRX1: NOD-like receptor family member X1
MAVS: Mitochondrial antiviral signaling
mTOR: Mammalian target of rapamycin; CVB3: Coxsackievirus B3
NF-κB: Nuclear factor κ-light-chain-enhancer of activated B cells
NOTCH1: Notch receptor 1
IFN-I: Activation of the type I interferon
FOXO3: Targeting Forkhead box O3
