# Supplementary figures and images for: Integrated analysis of microRNA and messenger RNA expression profiles reveals functional microRNA in infectious bovine rhinotracheitis virus-induced mitochondrial damage in Madin-Darby bovine kidney cells
Source: BMC Genomics. 2024 Feb 8;25:158. doi: 10.1186/s12864-024-10042-6 (PMC10851472; doi:10.1186/s12864-024-10042-6)

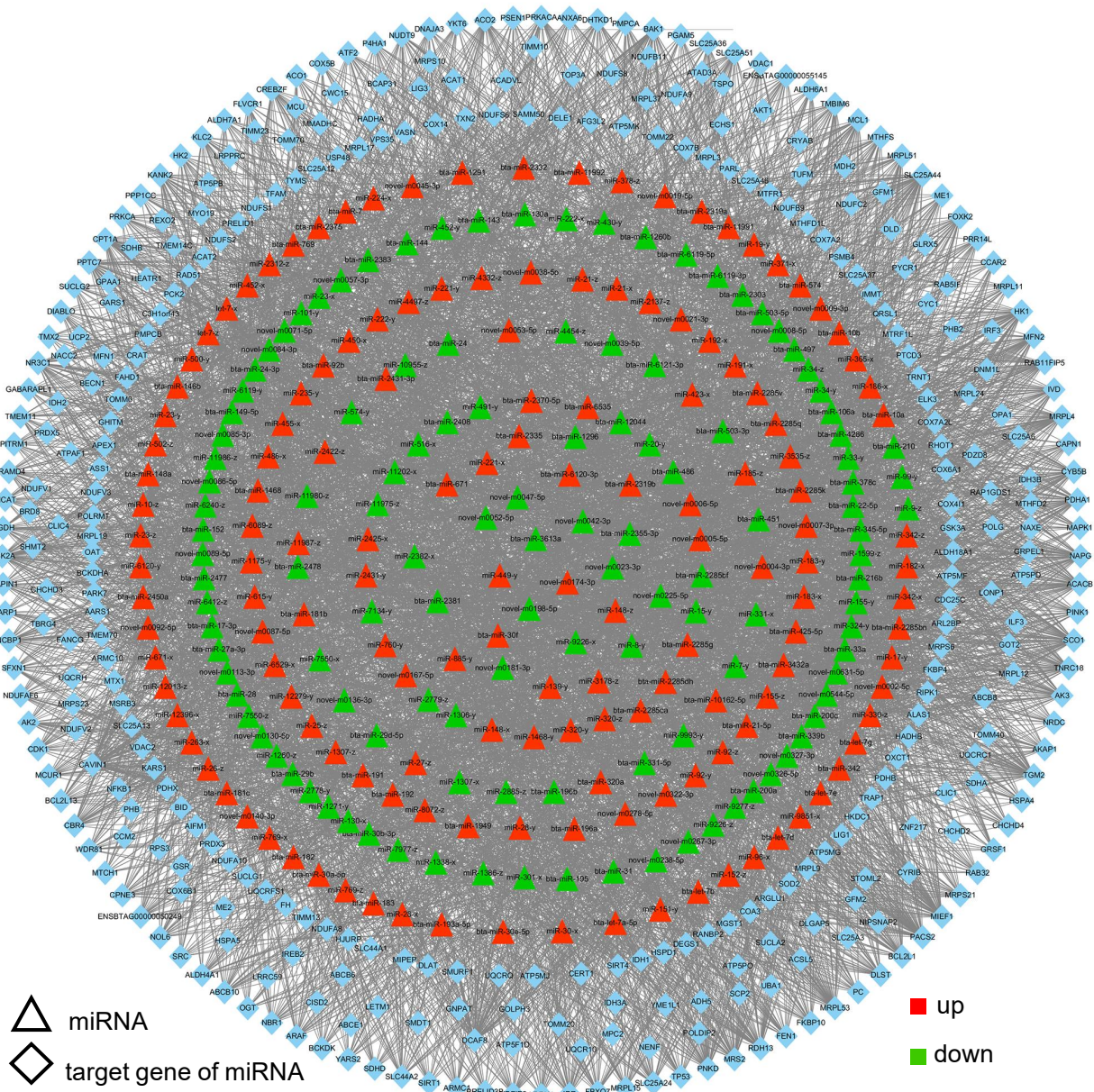

Supplement: Supplementary file 10 — Additional file 10: Figure S1. MiRNA-mitochondria-related target gene regulatory networks. [file 12864_2024_10042_MOESM10_ESM.pdf]
