# Supplementary material for: Integrated analysis of microRNA and messenger RNA expression profiles reveals functional microRNA in infectious bovine rhinotracheitis virus-induced mitochondrial damage in Madin-Darby bovine kidney cells
Source: BMC Genomics. 2024 Feb 8;25:158. doi: 10.1186/s12864-024-10042-6 (PMC10851472; doi:10.1186/s12864-024-10042-6)
Supplement: Supplementary file 14 — Additional file 14: Figure S1. MiRNA-mitochondria-related target gene regulatory networks. The triangles represent miRNA, the diamonds represent the target gene of the miRNAs, and the red and green shaded represent upregulated and downregulated miRNAs, respectively. [file 12864_2024_10042_MOESM14_ESM.docx]

**Figure S1. MiRNA-mitochondria-related target gene regulatory networks**.

The triangles represent miRNA, the diamonds represent the target gene of the miRNAs, and

the red and green shaded represent upregulated and downregulated miRNAs, respectively.
